# Within- and between-Breed Selection Signatures in the Original and Improved Valachian Sheep

**DOI:** 10.3390/ani12111346

**Published:** 2022-05-25

**Authors:** Mária Mészárosová, Gábor Mészáros, Nina Moravčíková, Ivan Pavlík, Milan Margetín, Radovan Kasarda

**Affiliations:** 1 Faculty of Agrobiology and Food Resources, Institute of Nutrition and Genomics, Slovak University of Agriculture in Nitra, Tr. A. Hlinku 2, 94976 Nitra, Slovakia; meszarosovamaria@gmail.com (M.M.); radovan.kasarda@uniag.sk (R.K.); 2 Department of Sustainable Agricultural Systems, Division of Livestock Sciences, University of Natural Resources and Life Sciences, Vienna, Gregor-Mendel-Straße 33, 1180 Vienna, Austria; gabor.meszaros@boku.ac.at; 3 Research Institute of Animal Production—NPPC Slovakia, Hlohovecká 2, 95141 Nitra—Lužianky, Slovakia; ivan.pavlik@vuzv.sk; 4 Faculty of Agrobiology and Food Resources, Institute of Animal Science, Slovak University of Agriculture in Nitra, Tr. A. Hlinku 2, 94976 Nitra, Slovakia; milan.margetin@uniag.sk

**Keywords:** selection sweep, quantitative trait loci, inbreeding, ROH islands, linkage disequilibrium, endangered populations

## Abstract

**Simple Summary:**

Each country has their own national breeds that are well adapted to the local climate and other conditions. Our study examines two national sheep breeds from Slovakia, the Original and the Improved Valachian sheep. The main goal of the study was to identify parts of their genome which contain genes enabling such adaptations. These are the so called selection signatures. Within the breed we look for genomic regions that were remarkably similar among a large proportion of genotyped individuals. The results point towards adaptation to high altitude pastures and resistance to parasites. The signatures of selection between the Original and Improved Valachian sheep focus on regions with striking difference in the same region of the genome between the two breeds. These results highlighted genes that improve the milk, meat and wool production, and their quality characteristics.

**Abstract:**

This study explored the genomic diversity and selection signatures in two Slovakian national breeds, the Original Valachian and the Improved Valachian sheep. As they are an important animal genetic resource within the country, but with decreasing population size, our aim is to identify potentially valuable genomic regions. A total of 97 sheep (18 male and 79 female) from the Original Valachian, and 69 sheep (25 male and 44 female) from the Improved Valachian populations were genotyped using the GeneSeek GGP Ovine 50 K chip. The inbreeding levels were assessed with runs of homozygosity (ROH). The selection signatures within breeds were identified based on the top 1% of most homozygous regions within the breed, the so-called ROH islands. The selection signatures between breeds were assessed based on variance in linkage disequilibrium. Overall, we have identified selection signatures with quantitative trait loci (QTL) and genes pointing towards all three production purposes of the Valachian sheep, milk, meat, and wool, including their quality characteristics. Another group with apparent large importance was the various traits related to health and resistance to parasites, which is well in line with the sturdy nature of this breed.

## 1. Introduction

The domestication of various livestock species is one of the greatest achievements of humankind. This process inherently resulted in permanent changes in the domesticated species, related to their morphological characteristics, behavior, and production levels. Parallel to the changes at the phenotype level, there were changes also occurring on the genomes. These detectable changes are called selection signatures, and they are of great interest to evolutionary biologists and livestock researchers alike. 

Sheep was one of the prime targets of domestication efforts, due to the wide variety of useful products, ability to adapt to different climatic conditions, and manageable size [1]. Subsequently, a large variety of breeds were created during the process of artificial selection, with specialized (or sometimes combined) production of wool, meat, and milk. With the widespread availability of the single nucleotide polymorphism (SNP) genotype data, it became possible to characterize the worldwide sheep populations [2]. Such characterization efforts identified the number of sheep breeds somewhere between 1000 [3] and 1400 [4]. 

The Original Valachian sheep a national, autochthonous breed from Slovakia. The small population of ca. 2900 animals is located in the northern mountainous regions, as it is well adapted to the harsh conditions of high-altitude pastures there. It was traditionally kept for combined triple-use: milk, meat and wool. The production is low, with ca. 80 kg milk per lactation, body weight of 35–55 kg, and average daily gain of 200 g until weaning. The Improved Valachian sheep was created to address these low production characteristics, while keeping the adaptation and climate resistance traits. A targeted and planned crossbreeding scheme was implemented between 1950s and 1980s, using Texel, Hampshire Down, Lincoln Longwool, and other breeds. The Improved Valachian sheep has higher milk production of ca. 100 kg per lactation, much higher body weight of 50 to 75 kg and average daily gain to weaning about 220 g.

Such diversity of breeds could evolve due to the wide variety of natural and artificial selection pressures on the populations. The selection itself leaves detectable changes on the genome caused by the so-called genetic hitchhiking effect [5]. Here, the selection on one gene with beneficial effect inevitably changes the frequencies of nearby neutral alleles. With consistent selection pressure and long enough time, the beneficial allele, along with the surrounding neutral alleles, spread throughout the population, reducing variability (thus increasing homozygosity) and strengthening linkage disequilibrium (LD) in the region. These signals could be then utilized to detect selection signatures to learn about the possible uniqueness of the populations.

One of the most widespread methods to detect the reduced variance in genomic regions of selection signatures is based on so-called runs of homozygosity (ROH) islands. The ROH are fully homozygous genomic segments that occur due to the relatedness of the two parents. Because of this assumption, they are often used to determine the inbreeding coefficient of animals [6]. However, from the evolutionary standpoint, they are often utilized to search for highly conserved regions. When the ROH segments overlap between a sizeable proportion of the population, this could indicate ongoing selection, thus the presence of beneficial genes. The method was used by Mastrangelo et al. [7], who identified the top 1% SNPs most commonly occurring in the homozygous regions as ROH islands in sheep. Based on the genomic coordinates of these regions, they have identified genes related to pigmentation, growth, milk, and carcass traits. In their earlier work Mastrangelo et al. [8] also used the ROH islands to identify genes with local adaptation in the Valle de Belice sheep. Additionally, Liu et al. [9] carried out a study, where they used ROH islands to identify signatures in five indigenous sheep breeds, based on the HD SNP BeadChip. The observed ROH pattern then helped to uncover the different population histories of the breeds. This application also demonstrates the wide scale of applicability of the method on finding interesting genomic regions in small, endangered breeds, which could be conserved and utilized in the future. Similarly, the ROH were utilized as the means of evaluating the diversity, and the ROH islands to identify selection signatures in South African sheep breeds [10].

Another possibility to explore the variation, and therefore the impact of selection on the genome is via patterns of LD. The LD is the population-wide correlation of nearby variants and characterizes the strength of connection between pairs of loci. The so-called LD blocks build up as a result of selection, via the genetic hitchhiking effect. In short, the allele at which the selection is acting is not inherited independently, but together with the loci that are nearby. If the selection pressure is strong enough, the allele is widespread in the population, together with its neighboring neutral alleles. This process results in a strong correlation, thus strong LD within this region. The differences in LD blocks between breeds than indicate differences in selection. This method was used to detect selection signatures in cattle [11,12,13], pigs [14] and horses [15]. Compared to other popular selection signature estimation methods, the studies using the variance in LD are sparse. Even more so in sheep, when even after our best efforts we could not find any suitable mention for this overview.

For our current study, we intend to fill the gap in the knowledge in genomic characterization of small indigenous breeds. We focus in particular on the exploration of diversity and selection signatures of the two Slovakian national sheep breeds, the Original Valachian and the Improved Valachian. They are an important animal genetic resource with diminishing population size; thus, identifying their characteristic and potentially valuable genomic regions is of great importance. Therefore, our aim in this paper is to explore the selection signatures within and between the Original and Improved Valachian sheep populations.

## 2. Materials and Methods

A total of 97 sheep (18 male and 79 female) from the Original Valachian, and 69 sheep (25 male and 44 female) from the Improved Valachian populations were genotyped using the GeneSeek GGP Ovine 50 K chip, with DNA obtained from hair follicles. The quality control was done using PLINK 1.9 [16], with the same thresholds for removal of SNPs and animals for both populations. Animals with more than 10% missing SNPs were removed. From the SNPs those with more than 10% missingness, below 1% minor allele frequency and not following the expected Hardy–Weinberg equilibrium distributions at *p* < 10^−7^ were excluded from the data set for the LD based selection signature analyses. The minor allele frequency threshold was not considered for run of homozygosity analyses. The SNPs from unplaced or sex chromosomes were removed for all analyses. After quality control, 96 animals and 38,236 SNPs remained for the Original Valachian sheep, and 68 animals and 39,240 SNPs were left for the Improved Valachian sheep population.

The inbreeding levels were computed using the software cgaTOH [17], based on ROH segments with minimum length of 1, 2, 4, 8 and 16 Mb. To minimize the occurrence of both false negative and false positive ROH segments, additional quality check was implemented based on Ferenčaković et al. [18]. In short, the number of false negative ROH segments were decreased by allowing certain number of missing, and in case of very long segments even heterozygous SNPs in the run. No missing or heterozygous SNPs were allowed for ROH with minimum length of 1 and 2 Mb, one missing and no heterozygous for ROH with minimum length of 4 Mb, two missing and no heterozygous for ROH with minimum length of 8 Mb, and four missing and one heterozygous for ROH with minimum length of 16 Mb. In addition, all ROH had to have at least 15 consecutive SNPs and no gaps larger than 1 Mb. The inbreeding coefficient for each sheep was computed as:

(1) $$F_{ROH}=\frac{\sum_{k}ROH_{k}}{L}$$

where $\underset{k}{\sum }ROH_{k}$ is the sum of the ROH length for each segment *k*, and *L* is the length of the autosomal genome covered by SNPs.

Further, the overlap between the ROH regions with minimum length of 1 Mb was examined for each breed. Such overlaps were called ROH islands, and were considered as selection signatures. The exact cut-off value for the ROH islands to be analyzed in the follow up was defined as the top 1% of most homozygous regions. The results were visualized using R, and the regions were compared to the sheep QTLdb (animalgenome.org, accessed on 20 October 2021) to determine their potential functions.

The linkage disequilibrium was computed as squared correlations using PLINK’s—r2 option, between each SNP within 1000 kb windows. The SNPs were ordered based on the inter marker distance, and the LD decay was visualized as the average within non-overlapping sliding windows of 5 SNPs.

In addition, the varLD software [19] was used to compare the LD patterns between the two breeds, in our case, those of the Original and Improved Valachian sheep. For this purpose, only the 37,788 SNPs were used that fulfilled the quality control criteria and were simultaneously present in both populations. The computation of standardized values for varLD scores, as well as the visualization, was performed in R, using the scripts provided by the varLD authors [19]. The signals above the top 1% of the standardized scores were highlighted as selection signatures between the two breeds, with further fine-tuning of the scales at the top 0.1% and 0.01%.

## 3. Results and Discussion

### 3.1. Inbreeding Levels

The inbreeding levels for the two populations are summarized in Table 1. Depending on the minimum length of ROH segments that were considered for the computation, the resulting genomic inbreeding coefficients consider common ancestors from various points in the past. The most recent inbreeding with common ancestors up to 3 generations was described considering the ROH segment length with minimum of 16 Mb. The inbreeding levels for larger time spans in the past were described by shorter segments. Here, the segment length with a minimum of 8, 4, 2 and 1 Mb described inbreeding levels to 6, 12, 25 and 50 generations in the past, respectively. Upon comparison of the two populations, the Improved Valachian sheep was much less inbred compared to the Original Valachian sheep population. These results indicate differences in management of both breeds, especially for the avoidance of recent inbreeding. While the inbreeding up to three generations is fairly low also for the Original Valachian sheep, it starts to be a concern when looking deeper in the pedigree of animals.

### 3.2. Within-Breed Selection Signatures Based on Run of Homozygosity Islands

**Figure 1:**
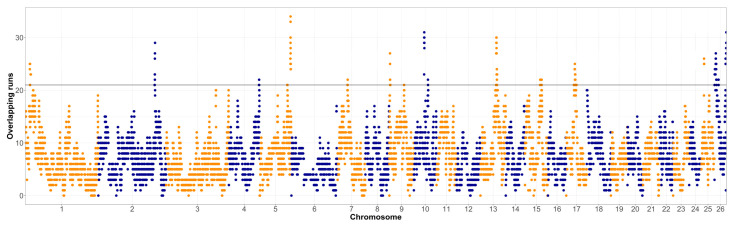
Runs of homozygosity islands in the Original Valachian sheep population (horizontal line denotes the top 1% of most homozygous regions, alternating colors distinguish markers on neighboring chromosomes).

**Figure 2:**
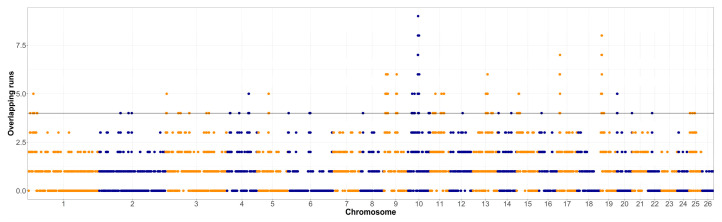
Runs of homozygosity islands in the Improved Valachian sheep population (horizontal line denotes the top 1% of most homozygous regions, alternating colors distinguish markers on neighboring chromosomes).

The overlap of ROH segments with minimum length of 1 Mb in both populations was assessed and visualized in Figure 1 and Figure 2. The shortest ROH segments were chosen for the assessment of the ROH islands, so even the older selection signatures could be detected. The top 1% of the genomic regions that were covered by ROH the most often were further investigated, first for the occurrence of relevant quantitative trait loci (QTL) for the Original Valachian (Supplementary Table S1) and the Improved Valachian sheep (Supplementary Table S2). Although there was no specific requirement for the top 1% most homozygous SNPs to be neighboring on the genome, the most homozygous SNPs still tended to be near each other. We refer to these homozygous regions for the purposes of identifying QTL content.

In both breeds, there were a number of QTLs related to the main production of the breed, meat, milk and wool. These were represented by QTLs for both the amount of production alone, but also quality parameters, such as fat and protein content of milk, pH and color traits of meat, and similar. Interestingly, in the Original Valachian sheep, there we fewer, but larger regions, also containing a range of adaptation and disease resistance traits, such as response to parasitic infections. In the Improved Valachian sheep the most homozygous regions were more scattered, and not as widespread within the population (the top region was in a ROH for just 8 animals). Such results could be expected due to the history of crossbreeding, with admixture breaking down longer, almost fixed genomic regions into shorter segments. The QTL content of these smaller segments throughout the genome was clearly related to production, mostly milk and meat, also including their quality parameters, but also QTLs related to parasite resistance and health traits. On chromosome 10, around the highest signal, there were a number of QTLs related to horn traits. This might be of significance and could merit a further study, due to the peculiar horn shape of the Valachian sheep. Another interesting region in the Improved Valachian sheep was a small region on chromosome 25 (12.4–12.6 Mb), with a large number of QTL related to wool production. Although wool is currently not of great importance, the previous breeding process placed some emphasis on the improvement of this traits as well, which might have left some detectable signatures behind.

The much lower inbreeding (Table 1) in the Improved Valachian sheep was also visible in the overall homozygosity in the genome. In both cases the ROH islands were de-fined as the top 1% of most homozygous regions in the genome. However, such threshold was clearly different between the Original and Improved Valachian sheep. While in the Original Valachian sheep the 1% threshold was at overlapping segments for 21 individuals, in Improved Valachian it was only for four individuals. Such clear differences were likely caused by recent breeding history. The Improved Valachian sheep was created using the Original Valachian sheep as its base, and the targeted crossbreeding activities were applied between the 1950s and 1980s. The goal was to keep to overall characteristics, the high altitude adaptation, good grazing abilities and disease resistance traits of the autochthonous Original Valachian sheep. At the same time, the production traits of wool, milk and meat were upgraded using other established breeds such as Texel, Hampshire Down, Lincoln Longwool, Leicester Longwool, and East Friesian. Such events brought in many new parental haplotypes, making it less probable even for shorter ROH to occur in the Improved Valachian sheep.

### 3.3. Linkage Disequilibrium

**Figure 3:**
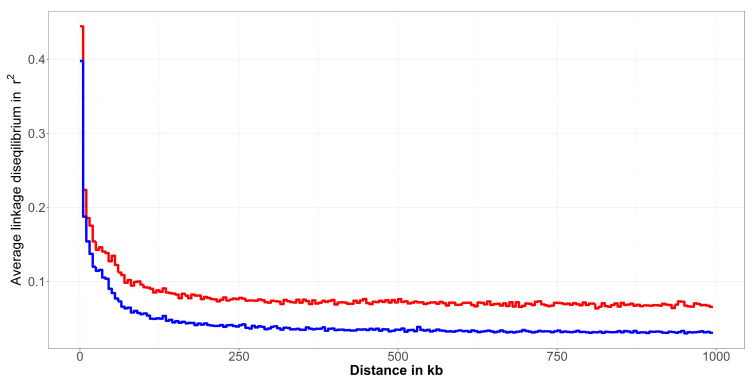
Linkage disequilibrium decay in the Original Valachian (red line) and Improved Valachian (blue line) sheep population.

Similarly to ROH results, the consequences of the applied breeding strategy were also visible for the comparison of LD decay in Figure 3. Additionally, here, the Improved Valachian sheep demonstrated a generally lower LD also in the case of SNPs in immediate vicinity. The LD values decrease much faster, to a considerably lower average values, compared to the Original Valachian sheep population. The LD extended to larger distances in the Original Valachian sheep, as it was undergoing selection for a much longer time period. In the Improved Valachian sheep the introduction of other breeds had a large effect on the LD structure in the population. It seems that not only the ancestral haplotypes in ROH were affected, but haplotypes across the genome were broken up. This breed improvement using other breeds largely affected the LD structure of the Improved Valachian sheep, with much lower LD across the genome, with the exception of SNPs very close to each other.

### 3.4. Between-Breed Selection Signatures Based on Variation of LD

**Figure 4:**
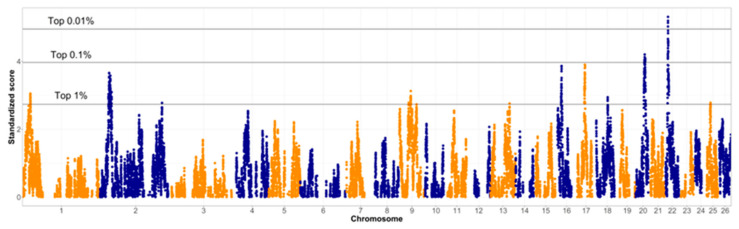
Standardized scores from the varLD indicate selection signatures between the Original and Improved Valachian sheep populations. Horizontal lines indicate highest results in percentage. Alternating colors distinguish markers on neighboring chromosomes.

After a breed-specific analysis of QTL functions in the identified regions, the two breeds were compared to identify regions where they differ from each other. Figure 4 shows the summary of these results. The differences between the correlation matrices were evaluated, and the regions that differ the most were highlighted as selection signatures. We provide visualization of thresholds for the top 1%, 0.1% and 0.01% of the signals in Figure 4, as well as Supplementary Figure S1. The top 1% of the signals included the 29.1 to 30.4 Mb region on chromosome 1, a wide region spanning from 31.7 to 43.4 Mb on chromosome 2, as well as a single SNP at 220.4 Mb of chromosome 2. Additionally, two single SNPs were appearing above the 1% threshold at chromosome 9 at 36.0 and 67.7 Mb, as well as a wider region between 42.7 and 44.9 Mb on chromosome 9. An additional single SNP was above the 1% threshold on chromosome 13, at 64.8 Mb. On chromosome 16, a wider region was identified, between 16.0 and 17.7 Mb. A somewhat wider region was found on chromosome 17, between 30.5 and 33.2 Mb. Other signals above the 1% threshold were found between 37.6 and 37.8 Mb of chromosome 18 and at 15.0 Mb of chromosome 25.

As for the top 0.1% highest signal, there was only one region on chromosome 20, between 29.9 and 32.7 Mb. There seems to be a gap in the signal between 30.9 and 31.9 Mb. This relatively wide signal does not have a pronounced peak, indicating that the whole genomic region could be of interest, rather than any specific point. The SNP indicating the highest signal within this region was located at 30.7 Mb. The top signal for the entire genome was found on chromosome 22. The top 0.1% region was found between 10.3 and 11.6 Mb. The region exceeding the 0.01% threshold was found between 10.8 and 11.0 Mb on chromosome 22.

In general, the more relaxed the thresholds were, the wider the section signatures became. This could be a possible reason why other studies, such as [11] and [14] evaluated only the top 0.1% and 0.01% signals. For both identified regions on chromosome 16 and 17, the top of these were close to the 0.1% threshold, thus their detailed exploration might be justified.

Our top signal directly overlaps with the genes *LIPA*, *IFIT2*, *IFIT3*, *IFIT5*, *SLC16A12* and three pseudogenes. The *LIPA* gene was found to be involved in the lipid mechanism and composition of lipoproteins [20], response to wounds and inflammations [21], but also in molecular genetic mechanisms affecting fecundity in sheep [22]. The family of *IFIT* genes was shown to have antiviral functions [23]. Similarly, in sheep, it was shown to influence parasite resistance in Merino sheep populations [24]. The *SLC16A12* gene was also shown to have various immune related functions, related to bacterial infections [25,26].

A large number of pseudogenes and protein coding genes were found in the other regions identified by the VarLD approach. Here, they are listed according to the chromosome number for the regions indicated above, and those with possible larger relevance are further described.

For chromosome 1, these protein coding genes were *USP24*, *PHF11*, *DHCR24*, *TTC22* and *TTC4*. The gene *PHF11* appears to inhibit replication of certain retroviruses [27] and play a role in activation of the T-cells in the immune system [28]. The *DHCR24* appears to have an important function related to the biosynthesis of cholesterol, but also involved in neurological disorders, such as the Alzheimer disease [29].

On chromosome 2, there was a very wide signal harboring a large number of protein coding genes, such as *DAPK1*, *FBP1*, *FBP2*, *CTSL*, *ISCA1*, *GOLM1*, *NTRK2*, *SLC28A3*, *RMI1*, *HNRNPK*, *KIF27*, *GKAP1*, *UBQLN1*, *FRMD3*, *IL11RA*, *ARID3C*, *DCTN3*, *DNAI1*, *C9orf24*, *NUDT2*, *KIF24*, *UBAP1*, *DCAF12*, *UBAP2*, *UBE2R2*, *NOL6*, *AQP3*, *AQP7*, *NFX1*, *BAG1*, *B4GALT1*, *DNAJA1*, *APTX*, *EPHX2*, *CHRNA2*, *PTK2B*, *DPYSL2*, *BNIP3L*, *PPP2R2A*, *EBF2*, *CDCA2*, *DOCK5*, *NEFL*, *NEFM*, *ADAM7*, *ADAM28*, *STC1*, *LOXL2*, *ENTPD4*, *RHOBTB2*, *PEBP4*, *EGR3*, *BIN3*, *SORBS3*, *DLIM2*, *PPP3CC*, *SLC39A14*, *PIWIL2*. Even though it is a very wide region with a high number of genes, there is a remarkable diversity in gene functions, which is relevant for productive, reproductive and health aspect of animals’ life. These associations were highlighted in a wide range of studies. Unfortunately, there were only a few studies done directly in sheep, but relevant studies were found in humans, but also in livestock, mostly cattle.

The *DAPK1* gene had an identified relationship to somatic cell score in cattle [30]. The *FBP1* gene plays an important role in the glucosynthesis [31], while the *FBP2* gene was shown to be related to milk productions traits [32], activating various metabolic processes in time of increased demands of high milk production [33]. The gene *CTSL* (cathepsin L) seems to be interesting from multiple angles, as it had been shown to influence carcass traits, production of milk [34] and reproductive traits [35]. The *ISCA1* was found to be associated with multiple mitochondrial dysfunctions [36]. According to a very recent study, the *NTRK2* was shown to be a novel candidate gene for litter size in sheep [37]. *RMI1* was shown to be associated with growth in sheep [38]. The *KIF27* gene was involved in fertility [39]. The gene *UBQLN1* is involved male fertility, more precisely in ubiquitination during spermatogenesis [40]. The *IL11RA* gene was also shown to be reproduction related, with its involvement in the placentation in multiple species [41]. *ARID3C* was involved in immunoglobulin gene transcription [42]. The *DNAI1* gene was identified to be relevant for spermatogenesis in cattle [43]. The *UBAP2* gene was found to be implicated in hypoxia and heat stress in sheep and goats [44]. In addition, in cattle it was in an LD block involved in wound healing, skin lesions, bone growth and mineralization [45]. The cold acclimatization is likely regulated by the *AQP3* and *APQ7* gene [46], also in this region. Such gene functions in the selection signatures in the Valachian sheep are well in line with its adaptation of high-altitude pastures. The *B4GALT1* gene was found to be associated with milk production in cattle [47]. The *DNAJA1* gene was highly expressed in both mammary gland and milk transcriptome, suggesting involvement in milk synthesis and production [48]. At the same time, it was found to be involved in meat tenderness in cattle [49]. Similarly, the nearby *APTX* gene was found in a study for marbling score in cattle [50]. The *PTK2B* gene was recently found to be involved in regulation of bovine mastitis [51]. The *DPYSL2* gene, also known as *CRMP2*, is a gene of major interest, as it could be involved in an extensive collection of neurodegenerative, sensory and motor neuron, and central disorders [52], which could be especially relevant for scrapie susceptibility in sheep. The *BNIP3L*, *PPP2R2A*, *CDCA2* and *NEFL* genes were related to reproductive traits, namely, to ovarian follicle development and with lambings out of season [53]. The *EBF2* was shown to be involved in regulation of body fat [54]. The *ADAM7* and *ADAM28* genes were previously found in genomic regions related to male fertility. In particular, *ADAM28* showed significant non-additive effects for sire conception rate in cattle [55]. The female reproduction via the uterine biology was influenced by the *STC1* gene [56]. The *LOXL2* gene was found to be related to meat production in cattle [12]. The *ENTPD4* gene seems to be influencing both milk production [57] and fertility [58]. Another gene from this region, which seems to be involved in multiple traits, was *PEBP4* which was found to simultaneously affect growth and fertility in cattle [59]. The gene *SORBS3*, *PPP3CC* and *PIWIL2* were located in a region associated with growth and wool traits in [60]. In addition, the *PPP3CC* gene was linked to heat tolerance in cattle [61], and the *PIWIL2* was associated with reproduction traits [62]. The *SLC39A14* gene was found to be associated with feed efficiency in cattle [63].

The second signal on chromosome was only a single significant SNP at 220.4 Mb. The genes around this SNP were: *TMBIM1* and *PNKD*, connected to meat color in cattle [64]; *USP37*, involved in regulation of reproduction in mice [65]; *VIL1*, related to meat tenderness [66]; *CTDSP1*, related to milk fatty acid synthesis in goats [67]; *SLC11A1*, with significant disease resistance related effects, in particular paratuberculosis, tuberculosis and brucellosis in a number of livestock species [68,69].

The next signal was on chromosome 9, around 36Mb, where the genes such as *LYN*, *PLAG1*, *RPS20* and *MOS* were located. From these the most important was the *PLAG1*, with a widely recognized effect on stature and growth traits in multiple species, including sheep [70,71]. The other genes in the region were also related to body size (*LYN*, *MOS*) but also fertility and dystocia (*RPS20*) [72]. The second signal indicated just by a single SNP on chromosome 9 was around 67.7Mb around the *SYBU* gene, which was related to milk fat synthesis, transport, and metabolism [73,74].

The wider region on chromosome 9 between 42.7 and 44.9 Mb contained protein coding genes such as *PDE7A*, *MTFR1*, *DNAJC5B*, *TRIM55*, *ADHFE1*, *CSPP1*, *COPS5*, *ARFGEF1*, and *CPA6*. From these genes, the most important were the *PDE7A* related to growth traits, through its significantly upregulation in lambs with muscle hypertrophy, callipyge [75]. The *TRIM55* gene was upregulated for beef tenderness, juiciness, and flavor [76]. The *COPS5* gene seems to be involved in multiple pathways, as it regulates the endometrial function in goats [77], was shown to be involved in mitochondrial functions [78], and carcass and meat quality traits [79]. The *ARFGEF1*, and *CPA6* were involved in weight and carcass traits in cattle [80].

On chromosome 13 at 64.8 Mb were the following genes: *SPAG4*, *NFS1*, and *ROMO1*. The *SPAG4* gene was shown to be connected to reproduction traits and spermatogenesis [81,82].

On the signal on chromosome 16, the *KIF2A* gene was located, which plays an important role during the meiosis, and its downregulation might interfere with the spermatogenesis [83]. One other gene of importance in this region was the *IPO11*, which was shown to be upregulated in mastitis [84], but also associated with displaced abomasum in cattle [85].

The signal on chromosome 17 was remarkably sparse, with almost no protein coding genes along the 3 Mb genomic region. The only gene in the region was the *FAT4* gene, associated with milk fat composition [86].

The signal on chromosome 18 at 37.8 Mb contains the gene *PRKD1*, associated with BMI and height in humans [87,88], but also associated with pubertal age in pigs [89].

Chromosome 25 contained a narrow signal indicated by two nearby SNPs at around 15 Mb. This region contained only two protein coding genes, *ANK3* and *CCDC6.* The *CCDC6* gene was of particular interest, as it was previously associated with body weight [59], but also in a gene network contributing to hypertrophy in callipyge skeletal muscle [90].

## 4. Conclusions

In the study, we carried out analysis of selection signatures within the Original and Improved Valachian sheep populations based on homozygosity and LD patterns on their genome. The results could be used in practice to improve the genomic characterization of the two breeds. The unique selection signatures for the respective breeds could serve as to clearly distinguish between the breeds on a genomic level, and aiding the decisions related to the conservation of genetic diversity. Overall, we have identified selection signatures with QTLs and genes pointing towards all three production purposes of the Valachian sheep, milk, meat and wool, including their quality characteristics. Another group with apparent large importance was the various traits related to health traits and resistance to parasites, which is well in line with the sturdy nature of this breed, kept mostly on high altitude pastures. More importantly, the genomic regions related to health and disease resistance, along with others pointing to various production traits, were also apparent in the Improved Valachian sheep. This indicates that the improvement process was a success that brought in increased productive capacity while retaining the adaptation characteristics of this Slovak traditional breed.

## Figures and Tables

**Table 1:** Summary statistics of the genomic inbreeding coefficients (*F*_ROH_) in the Original Valachian (OV) and Improved Valachian (IV) sheep.

	*F*_ROH_ (Up to 3 Generations)	*F*_ROH_ (Up to 6 Generations)	*F*_ROH_ (Up to 12 Generations)	*F*_ROH_ (Up to 25 Generations)	*F*_ROH_ (Up to 50 Generations)
OV	0.020 ± 0.026	0.038 ± 0.040	0.058 ± 0.052	0.068 ± 0.058	0.079 ± 0.064
IV	0.001 ± 0.006	0.003 ± 0.007	0.006 ± 0.010	0.008 ± 0.010	0.013 ± 0.011

## Data Availability

The data presented in this study are available on request from the corresponding author. The data are not publicly available due to ongoing research.

## Supplementary Materials

The following are available online at https://www.mdpi.com/article/10.3390/ani12111346/s1, Figure S1: Standardized VarLD scores per chromosome, Table S1: QTL content based on the Sheep QTLdb from the top 1% most homozygous regions in the Original Valachian sheep population, Table S2: QTL content based on the Sheep QTLdb from the top 1% most homozygous regions in the Improved Valachian sheep population
