# Supplementary material for: Within- and between-Breed Selection Signatures in the Original and Improved Valachian Sheep
Source: Animals (Basel). 2022 May 25;12(11):1346. doi: 10.3390/ani12111346 (PMC9179888; doi:10.3390/ani12111346)
Supplement: Supplementary file 1 [file animals-12-01346-s001.zip › SupplementaryTables.pdf]

Supplementary Table S1 QTL content based on the Sheep QTLdb from the top 1% most homozygous regions in the Original Valachian sheep population

Chromosome	Region start	Region end	QTL content trait and ontology
**1**	15990856	17348400	• muscle weight in carcass|PT:dressed carcass muscle weight<br>• Lean meat yield percentage|PT:dressed carcass muscle percentage<br>• Carcass fat percentage|PT:dressed carcass fat percentage
**1**	19367840	20833896	• muscle weight in carcass|PT:dressed carcass muscle weight<br>• Lean meat yield percentage|PT:dressed carcass muscle percentage<br>• Carcass fat percentage|PT:dressed carcass fat percentage<br>• Average daily gain|VT:postnatal growth trait|CMO:average daily body weight gain
**2**	211951580	213691445	• Milk fat percentage|VT:total milk fat amount|PT:milk fat content|CMO:milk fat percentage<br>• Meat eicosapentaenoic acid content|PT:meat fatty acid C20:5(n-3) content<br>• Subcutaneous fat thickness|VT:subcutaneous adipose thickness<br>• Hot carcass weight|PT:dressed carcass weight|CMO:carcass weight<br>• Body weight (slaughter)|VT:body mass|CMO:body weight<br>• Entropion<br>• Meat linolenic acid content|PT:meat fatty acid C18:3(n-3) content

			• Meat docosapentaenoic acid content|PT:meat fatty acid C22:5 content
**4**	119185515	122624922	• No QTLs
**5**	95613754	96465938	• Fecal egg count|VT:response to parasitic infection trait|CMO:fecal parasite egg count<br>• Clinical mastitis|CMO:disease incidence/prevalence measurement<br>• Body weight (birth)|VT:body mass|CMO:body weight
**5**	106977658	110570655	• No QTLs
**7**	33726396	34697173	• Longissimus muscle area|VT:longissimus thoracis muscle area<br>• Staple length|PT:wool staple length<br>• Primary fiber diameter coefficient of variance|PT:wool fiber trait
**9**	2625327	4521026	• Fecal egg count|VT:response to parasitic infection trait|CMO:fecal parasite egg count<br>• Useful yield content|PT:milk technological trait
**9**	59398870	59736336	• Fecal egg count|VT:response to parasitic infection trait|CMO:fecal parasite egg count<br>• Useful yield content|PT:milk technological trait
**10**	37484667	39294769	• Horns|CMO:horn number<br>• Somatic Cell Score|VT:milk somatic cell quantity|PT:milk somatic cell count|CMO:milk somatic cell score<br>• Fecal egg count|VT:response to parasitic infection trait|CMO:fecal parasite egg count<br>• Testes weight|VT:testis mass|CMO:testis weight<br>• fat weight in carcass|VT:white adipose mass|PT:dressed carcass fat weight|CMO:total body fat weight

			• Carcass bone percentage|PT:dressed carcass bone percentage<br>• Carcass fat percentage|PT:dressed carcass fat percentage<br>• Lean meat yield percentage|PT:dressed carcass muscle percentage
**10**	54113187	54733120	• Fecal egg count|VT:response to parasitic infection trait|CMO:fecal parasite egg count<br>• Testes weight|VT:testis mass|CMO:testis weight<br>• fat weight in carcass|VT:white adipose mass|PT:dressed carcass fat weight|CMO:total body fat weight<br>• Carcass bone percentage|PT:dressed carcass bone percentage<br>• Carcass fat percentage|PT:dressed carcass fat percentage<br>• Lean meat yield percentage|PT:dressed carcass muscle percentage
**13**	52466371	56712934	• muscle weight in carcass|PT:dressed carcass muscle weight
**15**	53596121	55100839	• Meat stearic acid content|PT:meat fatty acid C18:0 content<br>• Meat saturated fatty acid content|PT:meat saturated fatty acid content<br>• Meat oleic acid content|PT:meat fatty acid cis-9-C18:1 content<br>• Meat monounsaturated fatty acid content|PT:meat monounsaturated fatty acid content<br>• Fecal egg count|VT:response to parasitic infection trait|CMO:fecal parasite egg count<br>• Staple length|PT:wool staple length

**15**	59266868	59544484	• Carcass bone percentage∣PT:dressed carcass bone percentage<br>• muscle-to-bone ratio∣PT:dressed carcass muscle-to-bone ratio<br>• Carcass bone percentage∣PT:dressed carcass bone percentage<br>• muscle-to-bone ratio∣PT:dressed carcass muscle-to-bone ratio<br>• Staple length∣PT:wool staple length
**17**	27432170	32497516	• Average daily gain∣VT:postnatal growth trait∣CMO:average daily body weight gain<br>• Reproductive seasonality∣VT:female reproductive system physiology trait<br>• Fecal egg count∣VT:response to parasitic infection trait∣CMO:fecal parasite egg count<br>• Immunoglobulin A level∣VT:blood immunoglobulin A amount∣CMO:blood immunoglobulin A level<br>• Milk fat yield∣VT:total milk fat amount∣PT:milk fat yield∣CMO:milk fat yield<br>• Milk Yield∣VT:milk amount∣CMO:milk yield<br>• Milk fat yield∣VT:total milk fat amount∣PT:milk fat yield∣CMO:milk fat yield<br>• Breech Traits
**17**	35007054	37136604	• Milk Yield∣VT:milk amount∣CMO:milk yield<br>• Horn type
**25**	9640692	10798504	• Milk Yield {180d}<br>• Testes weight∣VT:testis mass∣CMO:testis weight<br>• Milk fat percentage∣VT:total milk fat amount∣PT:milk fat content∣CMO:milk fat percentage<br>• Useful yield content∣PT:milk technological trait<br>• Staple length∣PT:wool staple length<br>• Mean fiber diameter∣PT:wool fiber diameter

			• Primary fiber diameter coefficient of variance|PT:wool fiber trait<br>• Greasy fleece weight|PT:greasy fleece weight<br>• Staple length|PT:wool staple length
**26**	71911	7980544	• Average daily gain|VT:postnatal growth trait|CMO:average daily body weight gain<br>• Body weight (6 months)|VT:body mass|CMO:body weight<br>• Teat number|VT:nipple quantity|CMO:teat number<br>• Milk protein percentage|VT:milk protein amount|PT:milk protein content|CMO:milk protein percentage<br>• Muscle density<br>• Milk Yield|VT:milk amount|CMO:milk yield<br>• Entropion<br>• Milk fat yield {180d}<br>• muscle weight in carcass|PT:dressed carcass muscle weight<br>• Mean corpuscular volume|VT:erythrocyte size trait|CMO:mean corpuscular volume<br>• Reactivity to humans|VT:heterospecific interaction trait<br>• Vocalization behavior|VT:vocalization trait<br>• Footrot susceptibility|CMO:disease process measurement
**26**	14074241	15322098	• Average daily gain|VT:postnatal growth trait|CMO:average daily body weight gain<br>• Teat number|VT:nipple quantity|CMO:teat number<br>• Platelet count|VT:platelet quantity|CMO:platelet count<br>• Reproductive seasonality|VT:female reproductive system physiology trait

			• Total lambs born∣VT:offspring quantity<br>• Eggs per worm∣VT:response to parasitic infection trait<br>• Worm count∣VT:parasite quantity∣CMO:parasite count<br>• Change in hematocrit∣CMO:percent change in hematocrit<br>• muscle weight in carcass∣PT:dressed carcass muscle weight
**26**	41793640	42502511	• No QTLs
**26**	44812990	48258219	• No QTLs

Supplementary Table S2 QTL content based on the Sheep QTLdb from the top 1% most homozygous regions in the Improved Valachian sheep population

Chromosome	Region start	Region end	QTL content trait and ontology
**1**	7781921	8088015	• muscle weight in carcass|PT:dressed carcass muscle weight<br>• Body weight (birth)|VT:body mass|CMO:body weight
**1**	19646867	21716425	• muscle weight in carcass|PT:dressed carcass muscle weight<br>• Lean meat yield percentage|PT:dressed carcass muscle percentage<br>• Carcass fat percentage|PT:dressed carcass fat percentage<br>• Average daily gain|VT:postnatal growth trait|CMO:average daily body weight gain
	23669280	24454290	• muscle weight in carcass|PT:dressed carcass muscle weight<br>• Lean meat yield percentage|PT:dressed carcass muscle percentage<br>• Carcass fat percentage|PT:dressed carcass fat percentage
	35297095	36651483	• Bone weight in carcass|VT:bone mass|PT:dressed carcass bone weight|CMO:carcass skeletal weight<br>• muscle weight in carcass|PT:dressed carcass muscle weight<br>• Lean meat yield percentage|PT:dressed carcass muscle percentage<br>• Carcass fat percentage|PT:dressed carcass fat percentage<br>• muscle weight in carcass|PT:dressed carcass muscle weight
**2**	82487618	83602585	• Brown|VT:coat/hair pigmentation trait<br>• Somatic Cell Score|VT:milk somatic cell quantity|PT:milk somatic cell count|CMO:milk somatic cell score<br>• Teat number|VT:nipple quantity|CMO:teat number<br>• Subcutaneous fat thickness|VT:subcutaneous adipose thickness<br>• Trichostrongylus adult and larva count|VT:response to parasitic infection trait<br>• Meat color L*|PT:meat color reflectivity<br>• Meat color b*|PT:meat color yellowness<br>• Meat color a*|PT:meat color redness<br>• Ultimate pH|PT:meat pH

			• Meat eicosapentaenoic acid content|PT:meat fatty acid C20:5(n-3) content<br>• Meat linolenic acid content|PT:meat fatty acid C18:3(n-3) content<br>• Meat docosapentaenoic acid content|PT:meat fatty acid C22:5 content<br>• Milk protein percentage|VT:milk protein amount|PT:milk protein content|CMO:milk protein percentage<br>• Milk fat percentage|VT:total milk fat amount|PT:milk fat content|CMO:milk fat percentage<br>• Hot carcass weight|PT:dressed carcass weight|CMO:carcass weight<br>• Body weight (slaughter)|VT:body mass|CMO:body weight
**2**	113285547	122791886	• Many QTLs in this region (104 in total), including carcass and meat quality trait, as well as:<br>• Trichostrongylus adult and larva count|VT:response to parasitic infection trait<br>• Horn type
**3**	2188450	3440689	• Staple length|PT:wool staple length<br>• Haemonchus contortus FEC|VT:response to parasitic infection trait|CMO:fecal parasite egg count
**3**	46966552	47456534	• internal fat amount|VT:internal adipose amount<br>• Staple length|PT:wool staple length<br>• Body weight (56 weeks)|VT:body mass|CMO:body weight<br>• Milk Yield {180d}<br>• Milk fat yield {180d}<br>• Milk protein yield {180d}<br>• Milk fat percentage|VT:total milk fat amount|PT:milk fat content|CMO:milk fat percentage<br>• milk lactose yield|VT:milk lactose amount|PT:milk lactose yield
**3**	55897291	57254018	• Staple length|PT:wool staple length<br>• Milk Yield|VT:milk amount|CMO:milk yield<br>• internal fat amount|VT:internal adipose amount

			• Milk protein yield|VT:milk protein amount|PT:milk protein yield|CMO:milk protein yield<br>• milk lactose yield|VT:milk lactose amount|PT:milk lactose yield<br>• Meat conjugated linoleic acid content|PT:meat conjugated linoleic acid content
**3**	86157771	87276281	• Total lambs born|VT:offspring quantity<br>• internal fat amount|VT:internal adipose amount<br>• Staple length|PT:wool staple length<br>• Fecal egg count|VT:response to parasitic infection trait|CMO:fecal parasite egg count<br>• Change in hematocrit|CMO:percent change in hematocrit
**3**	153942575	154122080	• internal fat amount|VT:internal adipose amount<br>• Meat conjugated linoleic acid content|PT:meat conjugated linoleic acid content<br>• Change in eosinophil number|VT:eosinophil quantity<br>• Milk protein percentage|VT:milk protein amount|PT:milk protein content|CMO:milk protein percentage<br>• Body weight (birth)|VT:body mass|CMO:body weight<br>• Milk fat percentage|VT:total milk fat amount|PT:milk fat content|CMO:milk fat percentage
**3**	163959380	164099380	• internal fat amount|VT:internal adipose amount<br>• Body weight (birth)|VT:body mass|CMO:body weight<br>• Milk fat percentage|VT:total milk fat amount|PT:milk fat content|CMO:milk fat percentage<br>• Strongyle FEC|VT:response to parasitic infection trait|CMO:fecal parasite egg count
**4**	12240153	12434019	• Haemonchus contortus FEC|VT:response to parasitic infection trait|CMO:fecal parasite egg count<br>• Body weight|VT:body mass|CMO:body weight<br>• Primary fiber diameter coefficient of variance|PT:wool fiber trait
**4**	13291231	14306707	• Haemonchus contortus FEC|VT:response to parasitic infection trait|CMO:fecal parasite egg count

			• Body weight|VT:body mass|CMO:body weight<br>• Primary fiber diameter coefficient of variance|PT:wool fiber trait
**4**	51055680	51902709	• Primary fiber diameter coefficient of variance|PT:wool fiber trait
**4**	89890524	93309390	• Milk Yield|VT:milk amount|CMO:milk yield<br>• Milk fat percentage|VT:total milk fat amount|PT:milk fat content|CMO:milk fat percentage<br>• Body weight (weaning)|VT:body mass|CMO:body weight<br>• Average daily gain|VT:postnatal growth trait|CMO:average daily body weight gain<br>• Milk fat yield {180d}<br>• Milk Yield {180d}<br>• Primary fiber diameter coefficient of variance|PT:wool fiber trait
**5**	39012945	40917287	• Body weight (birth)|VT:body mass|CMO:body weight<br>• Fatty Acid Content>>Meat palmitoleic acid content|PT:meat fatty acid cis-9-C16:1 content<br>• Mean fiber diameter|PT:wool fiber diameter
**6**	2962737	3931207	• fat weight in carcass|VT:white adipose mass|PT:dressed carcass fat weight|CMO:total body fat weight<br>• fat weight in carcass|VT:white adipose mass|PT:dressed carcass fat weight|CMO:total body fat weight<br>• internal fat amount|VT:internal adipose amount<br>• Carcass fat percentage|PT:dressed carcass fat percentage<br>• Body weight (slaughter)|VT:body mass|CMO:body weight<br>• Lean meat yield percentage|PT:dressed carcass muscle percentage
**6**	39490694	40925809	• Many QTLs in this region (26 in total), including carcass and meat quality trait, as well as:<br>• Bone area<br>• Total bone|VT:bone mass<br>• Average daily gain|VT:postnatal growth trait|CMO:average daily body weight gain<br>• Fecal egg count|VT:response to parasitic infection trait|CMO:fecal parasite egg count

			• facial eczema susceptibility|CMO:disease process measurement<br>• Total fat area|PT:dressed carcass fat content<br>• Fat density<br>• Kleiber ratio
**8**	9474697	10196106	• Trichostrongylus adult and larva count|VT:response to parasitic infection trait<br>• Trichostrongylus adult and larva count|VT:response to parasitic infection trait<br>• Meat linolenic acid content|PT:meat fatty acid C18:3(n-3) content<br>• Meat omega-6 to omega-3 fatty acid ratio|PT:meat polyunsaturated fatty acid content<br>• internal fat amount|VT:internal adipose amount<br>• Fecal egg count|VT:response to parasitic infection trait|CMO:fecal parasite egg count
**9**	6582462	17078605	• Many QTLs in this region (18 in total), including, milk and growth traits, as well as:<br>• Maedi-Visna virus susceptibility|CMO:disease process measurement<br>• Fecal egg count|VT:response to parasitic infection trait|CMO:fecal parasite egg count<br>• Backfat at 12th/13th ribs|VT:subcutaneous adipose thickness
**9**	49343240	56307922	• Chest girth|VT:chest circumference|CMO:chest circumference<br>• Hot carcass weight|PT:dressed carcass weight|CMO:carcass weight<br>• Milk fat yield|VT:total milk fat amount|PT:milk fat yield|CMO:milk fat yield<br>• Entropion<br>• Longissimus muscle area|VT:longissimus thoracis muscle area<br>• Immunoglobulin A level|VT:blood immunoglobulin A amount|CMO:blood immunoglobulin A level<br>• Milk protein yield {180d}<br>• Milk fat yield {180d}

			• Milk Yield {180d}<br>• Haemonchus contortus FEC∣VT:response to parasitic infection trait∣CMO:fecal parasite egg count<br>• muscle weight in carcass∣PT:dressed carcass muscle weight
**10**	15667169	20149714	• Strongyle FEC∣VT:response to parasitic infection trait∣CMO:fecal parasite egg count<br>• Milk fat yield {180d}<br>• Milk protein yield {180d}<br>• Milk Yield {180d}<br>• Ear size∣VT:outer ear size trait<br>• Horns∣CMO:horn number<br>• fat weight in carcass∣VT:white adipose mass∣PT:dressed carcass fat weight∣CMO:total body fat weight<br>• Carcass bone percentage∣PT:dressed carcass bone percentage<br>• Carcass fat percentage∣PT:dressed carcass fat percentage<br>• Lean meat yield percentage∣PT:dressed carcass muscle percentage
**10**	25118745	27019110	• Wool crimp∣PT:wool crimp<br>• Horns∣CMO:horn number<br>• Immunoglobulin A level∣VT:blood immunoglobulin A amount∣CMO:blood immunoglobulin A level<br>• Total lambs born∣VT:offspring quantity<br>• Horn type<br>• Horn circumference∣CMO:horn circumference<br>• Tail fat deposition<br>• Fecal egg count∣VT:response to parasitic infection trait∣CMO:fecal parasite egg count<br>• Carcass bone percentage∣PT:dressed carcass bone percentage
**10**	36857185	44702797	• Horns∣CMO:horn number<br>• Horn length∣CMO:horn length<br>• Somatic Cell Score∣VT:milk somatic cell quantity∣PT:milk somatic cell count∣CMO:milk somatic cell score

			• Fecal egg count∣VT:response to parasitic infection trait∣CMO:fecal parasite egg count<br>• Testes weight∣VT:testis mass∣CMO:testis weight<br>• fat weight in carcass∣VT:white adipose mass∣PT:dressed carcass fat weight∣CMO:total body fat weight<br>• Carcass bone percentage∣PT:dressed carcass bone percentage<br>• Lean meat yield percentage∣PT:dressed carcass muscle percentage
**10**	83710317	87069386	• Fecal egg count∣VT:response to parasitic infection trait∣CMO:fecal parasite egg count<br>• fat weight in carcass∣VT:white adipose mass∣PT:dressed carcass fat weight∣CMO:total body fat weight<br>• Carcass fat percentage∣PT:dressed carcass fat percentage<br>• Lean meat yield percentage∣PT:dressed carcass muscle percentage<br>• Vocalization during arena test∣VT:vocalization trait
**10**	90098545	92139072	• No QTLs
**11**	11144325	11420213	• Trichostrongylus adult and larva count∣VT:response to parasitic infection trait<br>• milk conjugated linoleic acid content∣VT:milk conjugated linoleic acid amount∣PT:milk conjugated linoleic acid content<br>• milk conjugated linoleic acid content∣VT:milk conjugated linoleic acid amount∣PT:milk conjugated linoleic acid content<br>• internal fat amount∣VT:internal adipose amount<br>• Hot carcass weight∣PT:dressed carcass weight∣CMO:carcass weight<br>• Body weight (slaughter)∣VT:body mass∣CMO:body weight
**11**	14506105	15395509	• Trichostrongylus adult and larva count∣VT:response to parasitic infection trait<br>• Pleurisy<br>• internal fat amount∣VT:internal adipose amount<br>• Milk yield persistency∣VT:milk amount<br>• Milk protein yield∣VT:milk protein amount∣PT:milk protein yield∣CMO:milk protein yield

			• Hot carcass weight|PT:dressed carcass weight|CMO:carcass weight<br>• Body weight (slaughter)|VT:body mass|CMO:body weight<br>• milk polyunsaturated fatty acid content|VT:milk polyunsaturated fatty acid amount|PT:milk polyunsaturated fatty acid content|CMO:milk polyunsaturated fatty acid measurement
**11**	18526243	19856044	• Many QTLs in this region (18 in total), including, milk traits, as well as:<br>• Trichostrongylus adult and larva count|VT:response to parasitic infection trait<br>• internal fat amount|VT:internal adipose amount<br>• Tail fat deposition<br>• Total lambs born|VT:offspring quantity<br>• Haemonchus contortus resistance<br>• Total lambs born|VT:offspring quantity<br>• Hot carcass weight|PT:dressed carcass weight|CMO:carcass weight<br>• Body weight (slaughter)|VT:body mass|CMO:body weight
**11**	34831139	38038313	• Many QTLs in this region (23 in total), including, milk and carcass traits, as well as:<br>• Trichostrongylus adult and larva count|VT:response to parasitic infection trait<br>• Haemonchus contortus FEC|VT:response to parasitic infection trait|CMO:fecal parasite egg count<br>• Platelet count|VT:platelet quantity|CMO:platelet count<br>• Total lambs born|VT:offspring quantity<br>• Jaw length|VT:jaw morphology trait
**11**	43139005	45739629	• Many QTLs in this region (13 in total), including, milk and morphology traits, as well as:<br>• Trichostrongylus adult and larva count|VT:response to parasitic infection trait

			• Wool crimp∣PT:wool crimp<br>• Staple strength∣PT:wool staple strength<br>• Platelet count∣VT:platelet quantity∣CMO:platelet count<br>• Immunoglobulin A level∣VT:blood immunoglobulin A amount∣CMO:blood immunoglobulin A level
**12**	45956832	46503023	• Fecal egg count∣VT:response to parasitic infection trait∣CMO:fecal parasite egg count<br>• Haemonchus contortus FEC∣VT:response to parasitic infection trait∣CMO:fecal parasite egg count
**13**	45834290	50893239	• Milk Yield∣VT:milk amount∣CMO:milk yield<br>• muscle weight in carcass∣PT:dressed carcass muscle weight<br>• Tail fat deposition
**13**	62922996	63707268	• muscle weight in carcass∣PT:dressed carcass muscle weight<br>• Tail fat deposition
**13**	66151236	67411954	• Milk fat yield {180d}<br>• muscle weight in carcass∣PT:dressed carcass muscle weight
**14**	2782673	3026464	• Dressing percentage∣PT:dressing percentage<br>• Extension∣VT:coat/hair pigmentation trait<br>• Total bone∣VT:bone mass<br>• Bone weight in carcass∣VT:bone mass∣PT:dressed carcass bone weight∣CMO:carcass skeletal weight<br>• Nematodirus FEC∣VT:response to parasitic infection trait∣CMO:fecal parasite egg count
**14**	51809057	52277013	• Many QTLs in this region (13 in total), including, milk and weight traits, as well as:<br>• Nematodirus FEC∣VT:response to parasitic infection trait∣CMO:fecal parasite egg count
**15**	1898953	2039517	• No QTLs
**15**	7564286	13302798	• udder shape∣VT:udder morphology trait<br>• Red blood cell count∣VT:erythrocyte quantity∣CMO:red blood cell count<br>• Vocalization during arena test∣VT:vocalization trait

**16**	9976131	10273069	• Body weight (slaughter)ǀVT:body massǀCMO:body weight<br>• Milk YieldǀVT:milk amountǀCMO:milk yield<br>• Dressing percentageǀPT:dressing percentage<br>• Subcutaneous fat thicknessǀVT:subcutaneous adipose thickness<br>• Lean meat yield percentageǀPT:dressed carcass muscle percentage
**17**	7335961	9400383	• Reproductive seasonalityǀVT:female reproductive system physiology trait<br>• Fecal egg countǀVT:response to parasitic infection traitǀCMO:fecal parasite egg count
**19**	935502	3900264	• Total boneǀVT:bone mass
**19**	9406357	13116723	• Milk Yield {180d}<br>• Milk fat yield {180d}<br>• Ear sizeǀVT:outer ear size trait
**20**	477022	1994969	• Average daily gainǀVT:postnatal growth traitǀCMO:average daily body weight gain<br>• Milk protein percentageǀVT:milk protein amountǀPT:milk protein contentǀCMO:milk protein percentage
**20**	52827809	53289243	• No QTLs
**22**	15568265	15962167	• Somatic Cell ScoreǀVT:milk somatic cell quantityǀPT:milk somatic cell countǀCMO:milk somatic cell score
**25**	1965556	3079928	• Testes weightǀVT:testis massǀCMO:testis weight<br>• Staple lengthǀPT:wool staple length<br>• Mean fiber diameterǀPT:wool fiber diameter<br>• Primary fiber diameter coefficient of varianceǀPT:wool fiber trait<br>• Greasy fleece weightǀPT:greasy fleece weight<br>• Staple lengthǀPT:wool staple length
**25**	12422866	12608927	• Testes weightǀVT:testis massǀCMO:testis weight<br>• Milk fat percentageǀVT:total milk fat amountǀPT:milk fat contentǀCMO:milk fat percentage<br>• Useful yield contentǀPT:milk technological trait<br>• Staple lengthǀPT:wool staple length<br>• Mean fiber diameterǀPT:wool fiber diameter

			• Primary fiber diameter coefficient of variance|PT:wool fiber trait<br>• Greasy fleece weight|PT:greasy fleece weight
**25**	20634641	22724093	• Mean fiber diameter|PT:wool fiber diameter<br>• Testes weight|VT:testis mass|CMO:testis weight<br>• Nematodirus FEC|VT:response to parasitic infection trait|CMO:fecal parasite egg count<br>• Staple length|PT:wool staple length<br>• ean fiber diameter|PT:wool fiber diameter<br>• Primary fiber diameter coefficient of variance|PT:wool fiber trait
