# Supplementary figures and images for: Within- and between-Breed Selection Signatures in the Original and Improved Valachian Sheep
Source: Animals (Basel). 2022 May 25;12(11):1346. doi: 10.3390/ani12111346 (PMC9179888; doi:10.3390/ani12111346)

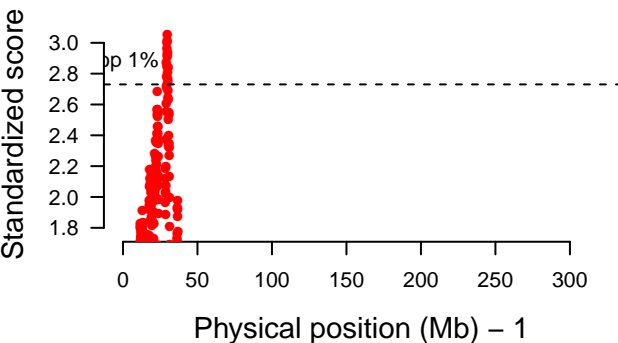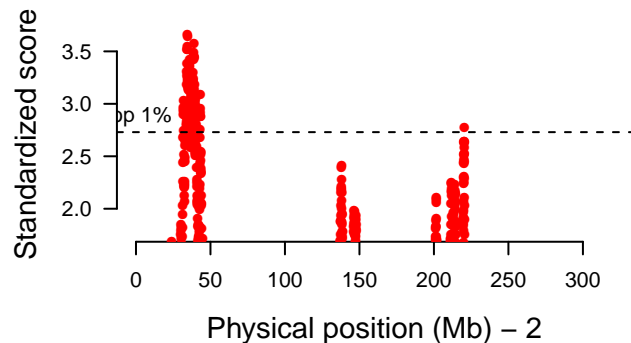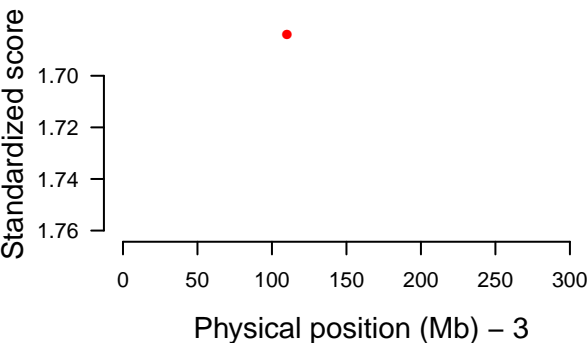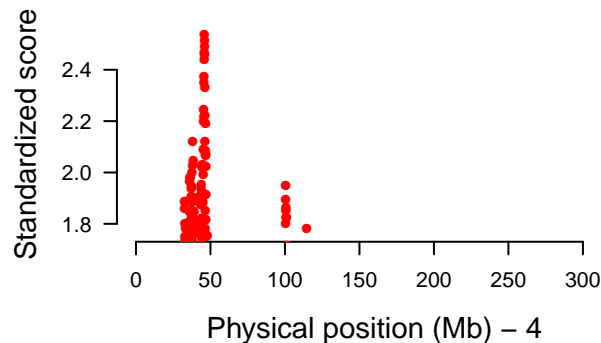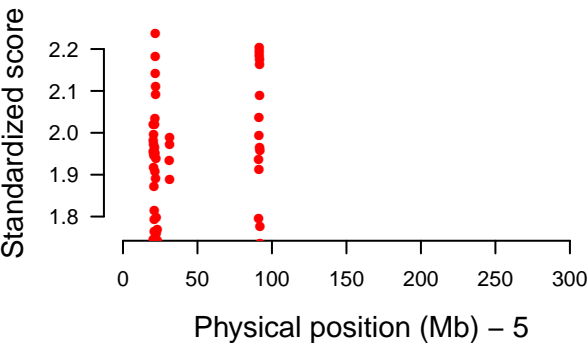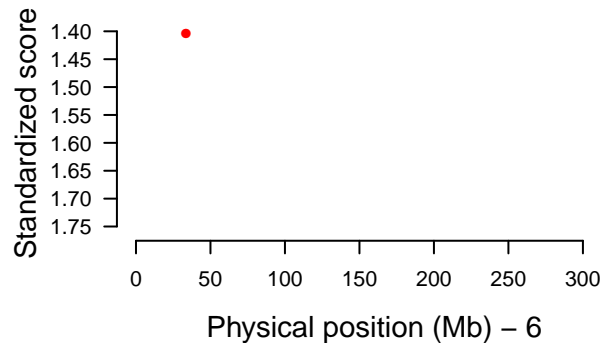

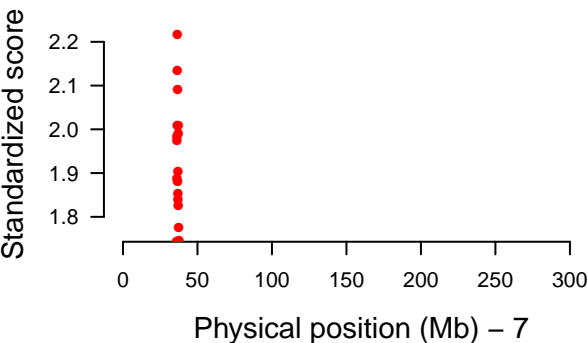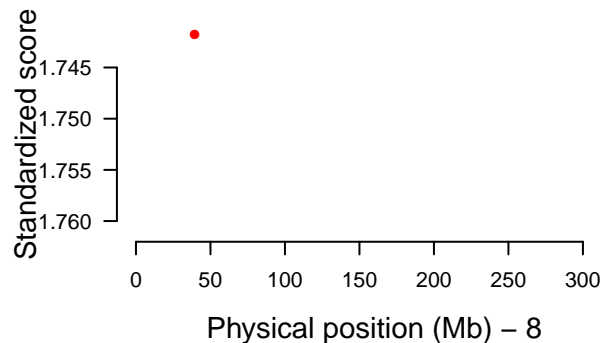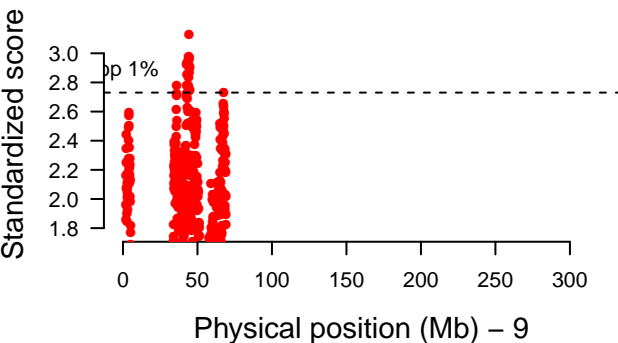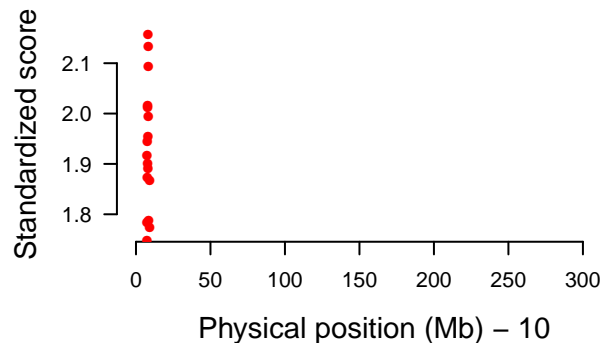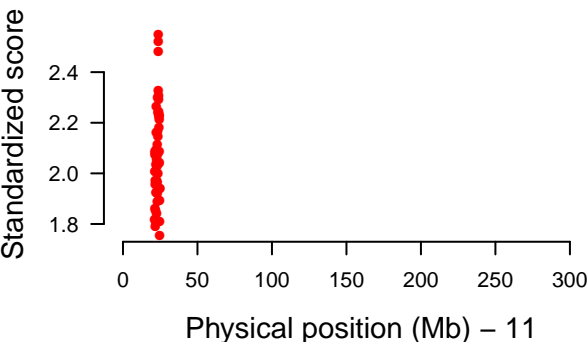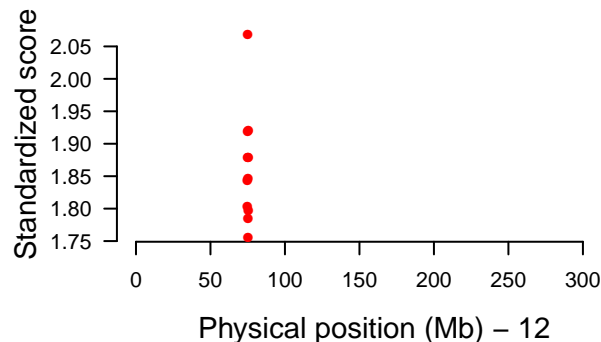

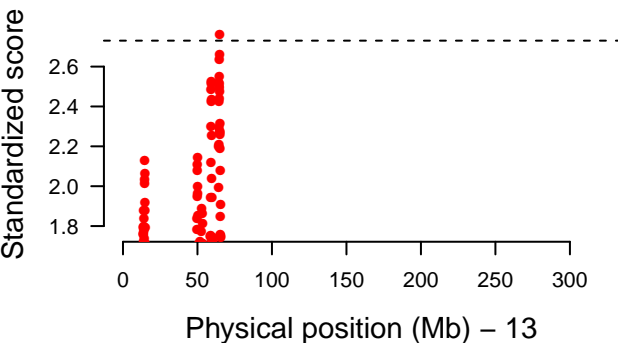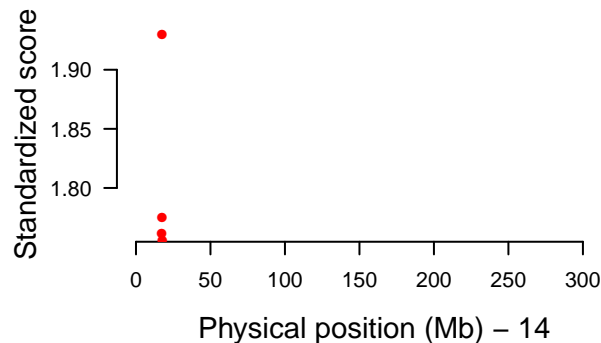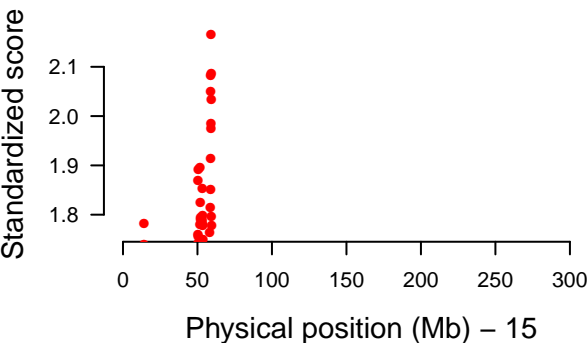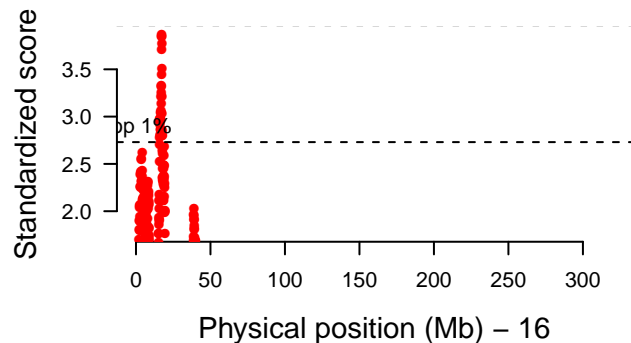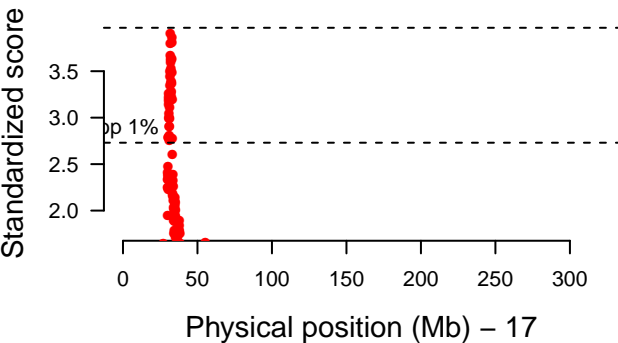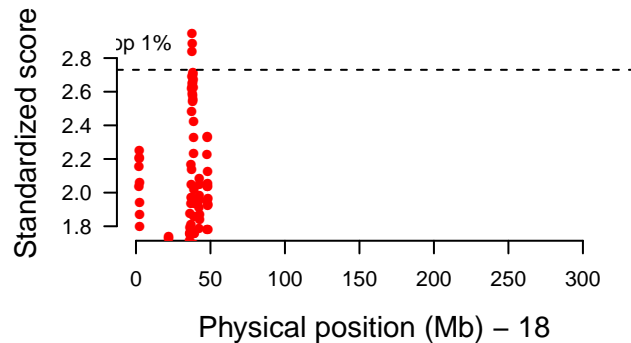

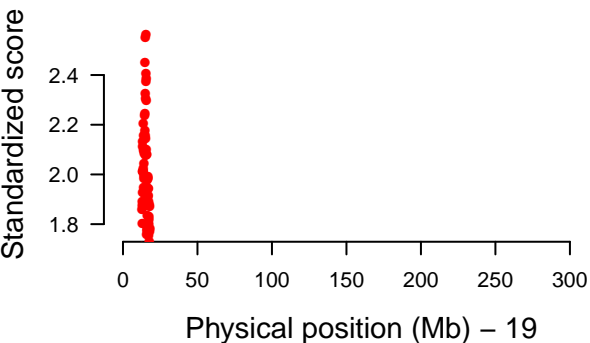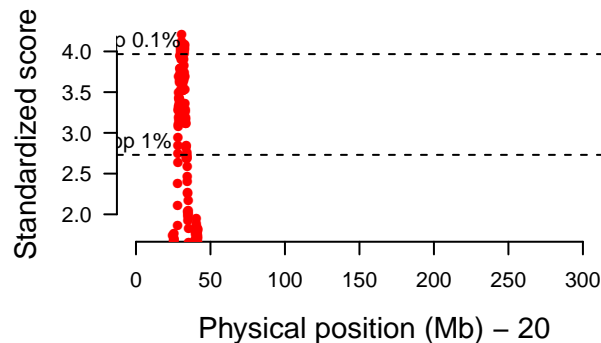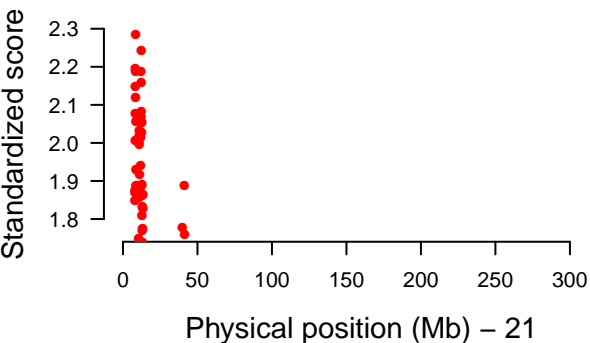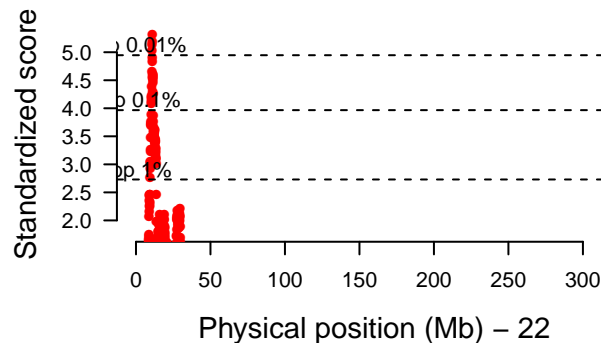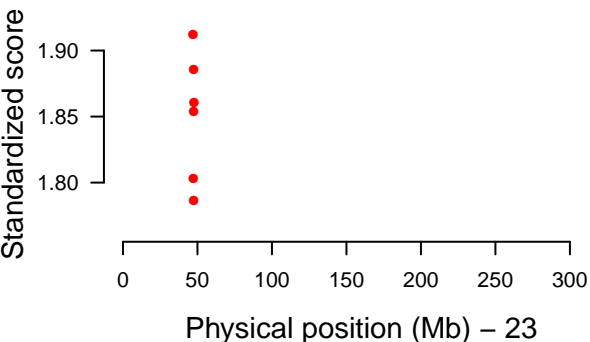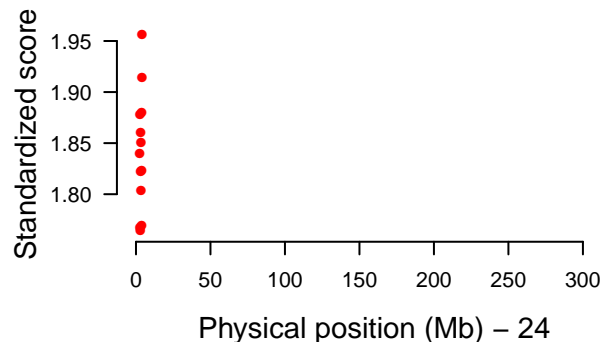

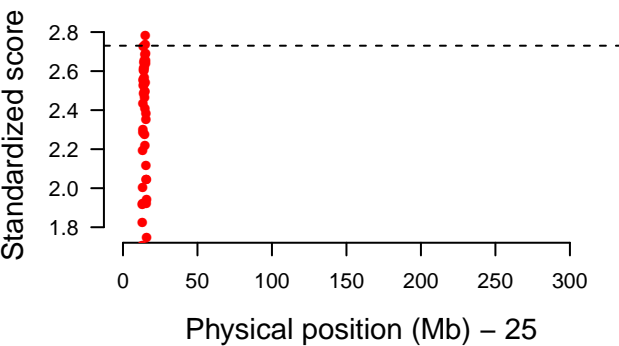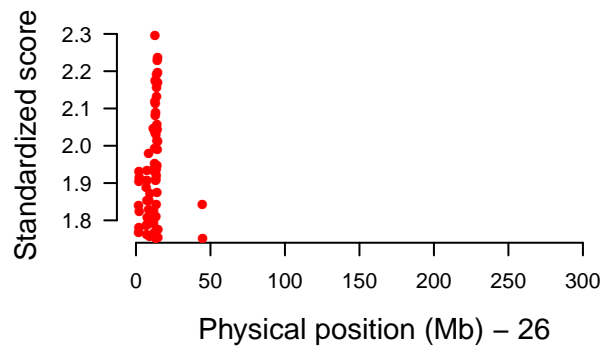

Supplement: Supplementary file 1 [file animals-12-01346-s001.zip › SupplementFigureS1_standardizedVarLdScoresPerChromosome.pdf]
